# A Compend of Diagnosis in Pathological Anatomy

**Published:** 1877-11

**Authors:** 


### BIBLIOGRAPHICAL.

A COMPEND OF DIAGNOSIS in Pathological Anatomy, with directions for making Post-Mortem Examinations. By Dr. Johannes Orth, First Assistant in Anatomy at the Pathological Institute in Berlin. Translated by Frederick Cheever Shattuck, M D., and George Kraus Sabine, M.D. Revised by Reginald Heber Fitz, M.D., etc. New York: Hurd & Houghton. 1878.

We have been permitted to look so far into the future as to take a glance at this, we presume, valuable book, dated next year. It reaches us so late that a perusal cannot be had in time for a notice of its merits in the November number of the JOURNAL. Having hastily scanned its contents, however, we are rather favorably impressed with the work, and promise ourself the benefit of a thorough reading.

